# In vitro assessment of mouse fetal abdominal aortic vascular function

**DOI:** 10.1152/ajpregu.00058.2014

**Published:** 2014-07-23

**Authors:** Lewis J. Renshall, Mark R. Dilworth, Susan L. Greenwood, Colin P. Sibley, Mark Wareing

**Affiliations:** ^1^Maternal and Fetal Health Research Centre, Institute of Human Development, Faculty of Medical and Human Sciences, University of Manchester, Manchester, United Kingdom; and; ^2^St. Mary's Hospital, Central Manchester University Hospitals National Health Service Foundation Trust, Manchester Academic Health Science Centre, Manchester, United Kingdom

**Keywords:** mouse, fetal, aorta, vascular, sildenafil citrate

## Abstract

Fetal growth restriction (FGR) affects 3–8% of human pregnancies. Mouse models have provided important etiological data on FGR; they permit the assessment of treatment strategies on the physiological function of both mother and her developing offspring. Our study aimed to *1*) develop a method to assess vascular function in fetal mice and *2*) as a proof of principle ascertain whether a high dose of sildenafil citrate (SC; Viagra) administered to the pregnant dam affected fetal vascular reactivity. We developed a wire myography methodology for evaluation of fetal vascular function in vitro using the placenta-specific insulin-like growth factor II (*Igf2*) knockout mouse (P0; a model of FGR). Vascular function was determined in abdominal aortas isolated from P0 and wild-type (WT) fetuses at embryonic day (E) 18.5 of gestation. A subset of dams received SC 0.8 mg/ml via drinking water from E12.5; data were compared with water-only controls. Using wire myography, we found that fetal aortic rings exhibited significant agonist-induced contraction, and endothelium-dependent and endothelium-independent relaxation. Sex-specific alterations in reactivity were noted in both strains. Maternal treatment with SC significantly attenuated endothelium-dependent and endothelium-independent relaxation of fetal aortic rings. Mouse fetal abdominal aortas reproducibly respond to vasoactive agents. Study of these vessels in mouse genetic models of pregnancy complications may *1*) help to delineate early signs of abnormal vascular reactivity and *2*) inform whether treatments given to the mother during pregnancy may impact upon fetal vascular function.

fetal growth restriction (FGR) is a major pregnancy problem affecting 8% of births in the United Kingdom (46). The etiology of FGR is multifaceted; maternal, fetal, and placental factors may influence fetal growth and development (21). A pathologically small size at birth also has major consequences for health, with increased risks of coronary heart disease, hypertension, and diabetes mellitus in adulthood. Despite these clinical implications, there is a dearth of effective treatments for affected pregnancies. Indeed, obstetrics has been described as a “pharma-free zone”; few drugs are under active development (10), partially as a result of concerns about detrimental effects of maternal treatments on the developing offspring.

As has been reviewed recently, genetically modified mouse models may provide ways to study FGR and find novel treatments (9). Human and mouse placentas are both hemochorial, although the latter exhibit extra trophoblastic layers and a labyrinthine, rather than villus, vascular architecture (5). Functionally, the placentas from the two species are similar: nutrient exchange and transport data suggest that passive permeability (34), transporter expression (2), and transporter activity (9, 16, 17) are generally comparable. Mouse models that exhibit FGR include the endothelial nitric oxide synthase (eNOS) knockout mouse (18, 43) and the placenta-specific *Igf2* knockout (P0) mouse (2, 16), in which 93% of P0 fetuses fall below the 5th percentile of C57BL6/J wild-type (WT) weights at term (8).

Vascular physiology in mouse pregnancy also appears similar to humans. Pregnant CD1 mouse uterine arteries exhibit characteristic low resistance and high-flow Doppler waveform pattern with high diastolic blood flow velocity (25). Furthermore, increased peak systolic and end-diastolic velocity and decreased uterine arterial resistance index in pregnant mice, compared with nonpregnant controls, are reminiscent of the altered hemodynamics of human pregnancy (25). Mouse umbilical arteries showed similar high-flow characteristics to those seen in humans (e.g., 24). Stanley et al. (39) described similar waveform patterns in uterine arteries of *Lepr*^*db/+*^ mice, which spontaneously develop gestational diabetes mellitus (GDM) (39); interestingly, reduced peak systolic and end-diastolic velocity and increased resistance indices in heterozygous mice suggest reduced uterine perfusion in diabetic animals.

These in vivo assessments, suggesting similar hemodynamics in mouse and human pregnancies, have been supported by in vitro studies. Decreased myogenic tone in pregnant vs. nonpregnant uterine arteries (44) and enhanced endothelium-dependent relaxation in pregnant mouse uterine arteries have been reported (3). Pulgar et al. (30) additionally noted increased uterine artery diameter and ANG II responses in pregnant vs. nonpregnant C57BL6/J mice (30). In pregnant C57BL6/J mice, we have extended these studies to assess fetal umbilical arterial and venous vascular reactivity (15). All of these reports support the in vivo evidence of increased uterine perfusion during mouse pregnancy, as occurs in women.

The rationale for using mouse models to study human pregnancy complications is, therefore, clear. Some studies have focused on diabetes in pregnancy [e.g., utilizing a streptozotocin-induced model (38) or the *Lepr*^*db/+*^ model of GDM (39, 40)]. Putative treatments have also been assessed, for example, by Spong et al. (35), who demonstrated reduced fetal demise by preadministration with glial protein-associated peptides in a pregnancy model of alcohol exposure. More recently, statin therapy for pregnancy complications has also been assessed; pravastatin administration potentially prevented placental damage and protected pregnancies in a model of recurrent spontaneous miscarriage (DBA/2-mated CBA/J mice) (31) and may ameliorate disease symptoms in a mouse model of preeclampsia (13). Indeed, our most recent studies have utilized this approach focusing on FGR, demonstrating improved fetal growth following administration of sildenafil citrate (SC; Viagra) to drinking water in both the P0 mouse model of FGR (6, 16) and the catechol-*O*-methyl transferase knockout mouse model of preeclampsia with FGR (37).

Dietary supplementation regimens have also received some attention. Pomegranate juice (in maternal drinking water) or administration of resveratrol (an active component pomegranate extract) has been suggested to be neuroprotective in a mouse model of hypoxic brain injury; offspring of treated pregnancies exposed to a hypoxic challenge 7 days postdelivery showed reduced brain tissue loss compared with controls (20, 45). In mice with streptozotocin-induced diabetes, Padmanabhan et al. (28) demonstrated that maternal dietary alpha-lipoic acid supplementation early in pregnancy improved pregnancy rates in diabetic animals; however, there was an increase in FGR and major congenital malformations in the treated group.

Thus, the potential of mouse models to assess targeted interventions for pregnancy complications is clear. However, no study has documented the effect of maternal treatment on the progeny's vascular function. The collection of such data is a key step to permit the development of successful therapies for pregnancy complications, such as FGR and preeclampsia. The aim of our current study was twofold. First, we wished to apply the wire myograph technique to study, for the first time, the reactivity of mouse fetal vessels at embryonic day (E) 18.5. This was achieved by utilizing wire myography to demonstrate measurable contraction and relaxation responses of fetal abdominal aortas from wild-type or P0 knockout fetuses, the latter as a well-characterized mouse model of FGR. Second, as a proof of principle, we wished to ascertain whether a pharmacological intervention targeted at the maternal vasculature would significantly affect fetal vascular reactivity (i.e., to determine the capability of the myography technique to detect an alteration in vascular function in fetal blood vessels). This was achieved by assessing abdominal aorta vascular reactivity from fetuses whose mother had been treated with a super-therapeutic dose of the PDE5 inhibitor SC during her pregnancy.

## MATERIALS AND METHODS

### 

#### Chemicals.

All chemicals were purchased from VWR International, (Lutterworth, UK) except NaCl and NaHCO_3_ (Fisher Scientific, Leicestershire, UK) and phenylephrine and acetylcholine (Sigma-Aldrich, Dorset, UK). Solutions were made daily and stored at 4°C.

#### Animals.

All experiments listed here were in accordance with the Animal Scientific Procedures Act (1986) and were covered by a UK Home Office Project Licence. Placenta-specific P0 knockout mice with deletion of the U2 exon within the *Igf2* gene (2, 6, 7, 16) were originally a kind gift from M. Constância and W. Reik (Babraham Institute, Cambridge, UK). Heterozygous males with the P0 deletion were mated with C57BL6/J female mice (WT; 6–10 wk of age; presence of copulation plug was considered E0.5; term was considered E19.5), which resulted in mixed litters consisting of both WT and P0 fetuses. P0 neonates are 25% smaller than their WT littermates and demonstrate increased behavioral anxiety in adulthood (22). Animals, housed in individually ventilated cages with nesting material, had access to standard pellet mouse chow (Beekay Rat and Mouse Diet; Bantin and Kingman; Hull, UK) and water ad libitum. Animals were maintained under constant 12:12-h light-dark cycle at 21–23°C.

#### Genotyping.

P0 mouse genotyping was performed as described previously using genomic DNA extracted from fetal tail tips (6). Sex genotyping was also performed on DNA extracted from WT and P0 mice fetal tail tips based on the method published by Kunieda et al. (14). The following primers were used to amplify the SRY sequence (male Y chromosome) and DXNds3 sequence (female X chromosome): SRY-forward 5′-TCTTAAACTCTGAAGAAGAGAC-3′ and SRY-reverse 5′-GTCTTGCCTGTATGTGATGG-3′; DXNds-forward 5′-GAGTGCCTCATCTATACTTACAG-3′ and DXNds-reverse 5′-TCTAGTTCATTGTTGATTAGTTGC-3′.

#### Fetal tissue collection and phenotypic measurements.

Pregnant female mice (*n* = 9) and fetuses (*n* = 43) were humanely euthanized by cervical dislocation [schedule 1 procedure in accordance with the UK Animals (Scientific Procedures) Act 1986] at E18.5 days gestation. A surgical laparotomy was performed, and the uterine horn was removed and placed into ice-cold tissue collection buffer. The intact uterine horn was pinned out as described previously (15). Individual pups were identified, dissected free from surrounding tissues, and placed into a sampling dish; tissues were kept moist with physiological salt solution (PSS) in mM: 119 NaCl, 25 NaHCO_3_, 4.69 KCl, 2.4 MgSO_4_, 1.6 CaCl_2_, 1.18 KH_2_PO_4_, 6.05 glucose, 0.034 EDTA (free acid) at pH 7.4.

For each individual, the pup with its corresponding placenta was blotted and weighed. On each pup, a surgical laparotomy was performed to expose the abdominal aorta from the level of the renal arteries to the femoral arterial bifurcation (∼2–3-mm lengths). Upon completion of the dissection, aortas were mounted onto a Danish Myo Technologies 610 M wire myograph, as described previously (4). Vessels were equilibrated in 6 ml PSS, warmed to 37°C, and gassed with 5% O_2_/5% CO_2_/balance N_2_.

#### Contraction-relaxation responses of isolated fetal abdominal aortas.

In preliminary experiments, abdominal aortas were normalized to 0.9 of L_13.3kPa_, but this level of applied stretch induced damage to the vessels, making reliable vascular function assessment impossible (data not shown). Aortas were, therefore, normalized to 0.9 of L_5.1kPa_ (4) and equilibrated for 20 min. Postequilibration, aortas were exposed to two separate exposures to a depolarizing solution (KPSS in mM: 12.45 NaCl, 25 NaHCO_3_, 120 KCl, 2.4 MgSO_4_, 1.6 CaCl_2_, 1.18 KH_2_PO_4_, 6.05 glucose, 0.034 EDTA (free acid) at pH 7.4). After washing with PSS, aortic contraction was assessed with 10^−5^ M phenylephrine (PE) and the thromboxane mimetic U46619 (10^−10^−2 × 10^−6^ M; 2-min intervals). After washing with PSS, blood vessels were precontracted with an effective concentration (EC) EC_80_ dose of U46619 and exposed to ACh (10^−10^–10^−5^ M). After washing with PSS, vessels were precontracted with an EC_80_ dose of U46619 and exposed to the nitric oxide donor sodium nitroprusside (SNP; 10^−10^–10^−5^ M).

#### Effect of maternally targeted SC treatment on fetal vascular function.

In a second series of experiments, pregnant female mice (*n* = 9) were treated with SC (Pfizer, Sandwich, UK). Treatment (0.8 mg/ml in drinking water) was commenced on E12.5 (the gestation at which blood is first supplied to the placenta) and continued until E18.5, with a fresh bottle made up at E15.5. As this was a proof of principle to assess the sensitivity of the myography technique, 0.8 mg/ml SC was chosen (i.e., twice the therapeutic dose utilized in our previous study) to produce a dose of SC closer to 100 mg·kg^−1^·day^−1^ (given that P0 mice drink on average of 3–4 ml of water per day (6). At E18.5, dams were humanely euthanized, pup and placenta measurements and tissues were collected as described above. Myography data were collected from individual fetal abdominal aortas (*n* = 31), as described above (*Contraction-relaxation responses of isolated fetal abdominal aortas*).

#### Data analysis.

Myography data from male and female pups was analyzed separately to determine whether sex differences existed for contraction and relaxation data. Contraction data were calculated as active effective pressure using the following equation: Active effective pressure = (wall tension/2π)/vessel internal circumference.

Relaxation data were expressed as a percentage of EC_80_ precontraction to U46619. Data are expressed as means ± SE (unless noted) with *n* equal to number of fetuses. Myodata 2.02 (Myonic Software, National Instruments, Austin, TX), Excel and GraphPad Prism version 5.0 (GraphPad Software, San Diego, CA) were used to analyze the data. An assessment of whether data were normally distributed was performed using the Kolmogorov-Smirnov normality test prior to statistical analysis. Vascular diameter, maximal contraction, and basal tone data were not normally distributed and were, therefore, analyzed using the Kruskal-Wallis test. Dose-response curves and fetal phenotypic measurements were compared using two-way ANOVA followed by a Bonferroni post hoc test where appropriate. ECs were calculated for U46619, ACh, and SNP dose-response curves. For U46619, the Hill slope was set at 1 and the minima of the curve was constrained to equal 0. For ACh and SNP, the Hill slope was set at −1, and the maximum value of the curve was constrained to equal 0% residual contraction/100% relaxation. EC values were compared using Mann-Whitney *U*-test. Vessels that did not conform to classical dose-response curves were not included in the analysis. *P* < 0.05 was taken to be indicative of statistical significance.

## RESULTS

### 

#### Contraction/relaxation responses of isolated fetal abdominal aortas (maternal water drinking controls).

There was no significant difference in abdominal aortic diameter between the four control groups (Kruskal-Wallis test; *P* > 0.05; Table 1). Abdominal aortic basal tone (0.9 of L_5.1kPa_ normalization) was not significantly different between the four experimental groups (Kruskal-Wallis test; *P* > 0.05; Table 1); these data corresponded to a basal tone for the control group of 33 [11 − 67] mmHg [median (range: min to max); *n* = 43].

**Table 1.** Vessel diameter, basal tone, and contraction data for fetal abdominal aortas

				Contraction, kPa		
Maternal Treatment	Fetal ID (*n*)	AA Diameter, μm	Basal Tone, kPa	KPSS	PE	U46619
Control	WT Male (11)	668 [552 − 737]	4.4 [3.0 − 6.9]	0.34 [0.19 − 0.51]	0.18 [0.03 − 0.45]	1.13 [0.42 − 2.10]^a^
	WT Female (11)	650 [560 − 717]	4.5 [2.9 − 8.9]	0.35 [0.07 − 0.79]	0.25 [0.03 − 0.60]	0.80 [0.57 − 1.30]^a^
	P0 Male (10)	696 [592 − 798]	4.3 [1.5 − 5.9]	0.32 [0.10 − 0.96]	0.10 [0.00 − 0.30]	0.80 [0.16 − 1.83]^a^
	P0 Female (11)	688 [629 − 882]	4.5 [1.6 − 6.6]	0.35 [0.12 − 0.60]	0.22 [0.02 − 0.48]	0.82 [0.32 − 1.56]^a^
Viagra (SC)	WT Male (10)	715 [575 − 794]	4.9 [4.0 − 6.9]	0.31 [0.06 − 0.68]	0.14 [0.00 − 0.30]	1.04 [0.10 − 1.80]^b^
	WT Female (7)	717 [499 − 989]	5.3 [3.9 − 8.9]	0.26 [0.05 − 0.43]	0.11 [0.03 − 0.21]	0.90 [0.26 − 1.70]^b^
	P0 Male (6)	668 [575 − 775]	3.9 [2.9 − 7.2]	0.52 [0.04 − 1.67]	0.12 [0.05 − 0.25]	1.31 [0.24 − 3.69]^b^
	P0 Female (8)	757 [639 − 1025]	3.7 [2.2 − 5.0]	0.30 [0.05 − 0.46]	0.09 [0.00 − 0.16]	0.90 [0.34 − 1.28]^b^

All data are median [min − max]. AA, abdominal aortas; KPSS, high potassium depolarizing solution; PE, 10^−5^ M phenylephrine. U46619 was administered at 2 × 10^−6^ M. Number of animals appear in parenthesis. Significantly increased contraction with U46619 vs. PE in control

a and SC ^b^ data (Kruskal-Wallis test; *P* < 0.05).

Contraction to each agonist (KPSS; 10^−5^ M PE; 2 × 10^−6^ M U46619) was not significantly different between each control group (Kruskal-Wallis test; *P* > 0.05; Table 1). U46619 elicited greater agonist-induced contraction compared with that with PE (Kruskal-Wallis test; *P* < 0.05; Table 1).

**Fig. 1.**
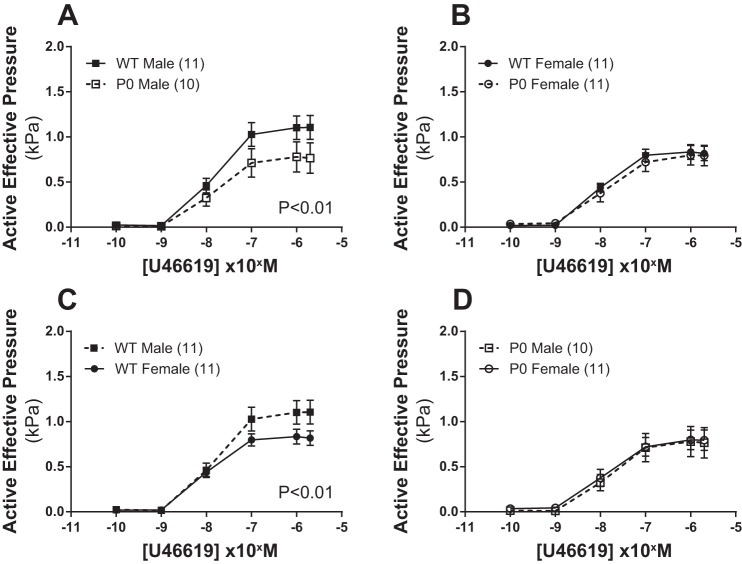
Contraction of fetal abdominal aortas in response to increasing doses of U46619. *A–D*: all data are expressed as means ± SE (*n* = number of animals). U46619 dose-response curves were compared using two-way ANOVA followed by a Bonferroni post hoc test where appropriate. ■ denotes WT male, while ● denotes WT female, □ denotes P0 male, and ○ denotes P0 females.

Dose-response curves for U46619-induced contraction are shown in Fig. 1. U46619-induced contraction was higher in WT male vs. WT female mice (*P* < 0.01; two-way ANOVA) but not in P0 male vs. P0 female mice (*P* > 0.05; two-way ANOVA). When pup sex was compared across genotype, U46619-induced contraction in male mice was greater in WT vs. P0 animals (*P* < 0.01; two-way ANOVA), but this sex effect was not seen in WT vs. P0 females (*P* > 0.05; two-way ANOVA).

**Fig. 2.**
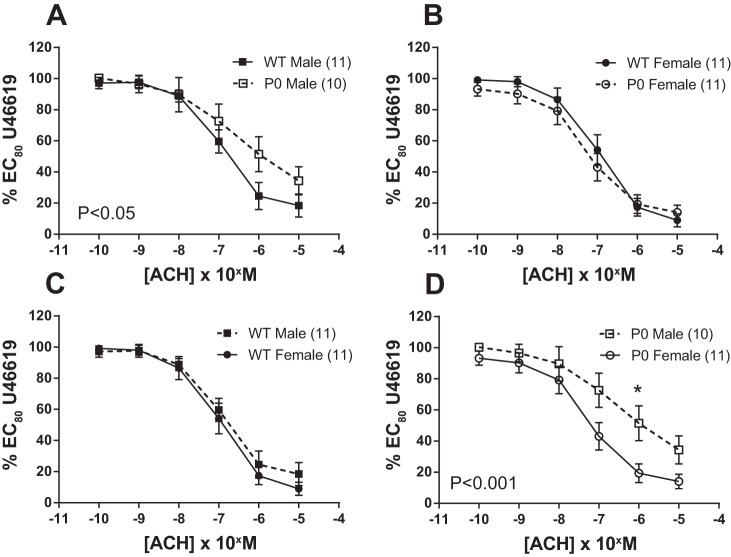
Relaxation of fetal abdominal aortas in response to increasing doses of ACh. *A–D*: arteries were precontracted with EC_80_ dose of U46619. ACh dose-response curves were compared using two-way ANOVA followed by a Bonferroni post hoc test where appropriate. All data are expressed as means ± SE (*n* = number of animals). ■ denotes WT male, while ● denotes WT female, □ denotes P0 male, and ○ denotes P0 females. Asterisks denote level of significance using Bonferroni post hoc test. *P < 0.05.

**Fig. 3.**
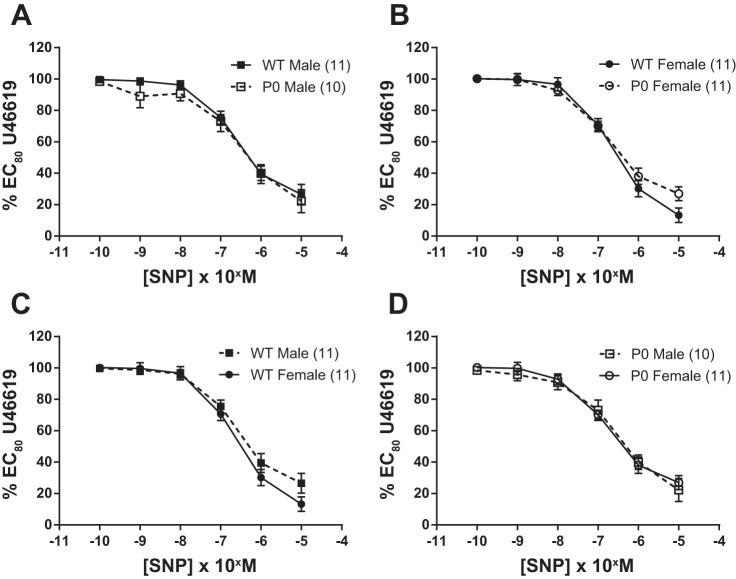
Relaxation of fetal abdominal aortas in response to increasing doses of SNP. *A–D*: arteries were precontracted with an EC_80_ concentration of U46619. SNP dose-response curves were compared using two-way ANOVA followed by a Bonferroni post hoc test where appropriate. All data are expressed as means ± SE (*n* = number of animals). ■ denotes WT male, while ● denotes WT female, □ denotes P0 male, and ○ denotes P0 females.

Both ACh (endothelium-dependent; Fig. 2) and SNP (endothelium-independent; Fig. 3) elicited significant relaxation of fetal abdominal aortas precontracted with an EC_80_ dose of U46619.

ACh-induced relaxation was similar in WT male vs. WT female mice (*P* > 0.05; two-way ANOVA) but was significantly blunted in P0 male vs. P0 female mice (*P* < 0.001; two-way ANOVA; Fig. 2). When sex of the pups was compared across genotype, ACh-induced relaxation was significantly blunted in P0 vs. WT male mice (*P* < 0.05; two-way ANOVA); however, in female mice, relaxation was similar comparing P0 and WT animals (*P* > 0.05; two-way ANOVA; Fig. 2).

SNP-induced relaxation was similar in WT male vs. WT female mice (*P* > 0.05; two-way ANOVA) and was not significantly different in P0 male vs. P0 female mice (*P* > 0.05; two-way ANOVA; Fig. 3). When sex of the pups was compared across genotype, SNP-induced relaxation was not significantly altered (*P* > 0.05; two-way ANOVA) in P0 vs. WT male mice or in P0 vs. WT female mice (*P* > 0.05; two-way ANOVA; Fig. 3). There were no significant differences between groups in the effective concentration of ACh and SNP required to produce 20, 50, and 80% relaxation (*P* > 0.05; Mann-Whitney *U*-test; Table 2). There were no significant differences in maximum responses (*V*_max_) for U46119, ACh and SNP (*P* < 0.05, Mann-Whitney *U*-test; data not shown).

**Table 2.** Fetal AA sensitivity to U46619, ACh, and SNP

Maternal Treatment	Agonist	Effective concentration	WT Male	WT Female	P0 Male	P0 Female
Control	U46619	EC_20_	4.5 ± 0.9 (11)	2.7 ± 0.5 (11)	4.5 ± 0.9 (10)	4.7 ± 1.3 (11)
		EC_50_	18.9 ± 3.6 (11)	10.8 ± 1.9 (11)	17.9 ± 3.6 (10)	18.9 ± 5.0 (11)
		EC_80_	49.0 ± 10.7 (11)	43.4 ± 7.7 (11)	71.7 ± 14.6 (10)	75.6 ± 19.9 (11)
	ACH	EC_20_	75.2 ± 39.1 (11)^A^	56.1 ± 21.8 (11)	42.2 ± 13.7 (6)	28.7 ± 12.0 (11)^B^
		EC_50_	300 ± 156 (11)^A^	224 ± 87.3 (11)	169 ± 54.8 (6)	115 ± 48.0 (11)^B^
		EC_80_	515 ± 101 (11)	844 ± 300 (11)	858 ± 253 (6)	397 ± 155 (11)^C^
	SNP	EC_20_	60.1 ± 8.6 (11)	60.2 ± 10.7 (11)	104 ± 31.5 (9)	40.1 ± 4.8 (11)^D^
		EC_50_	240 ± 34.5 (11)	241 ± 42.6 (11)	417 ± 126 (9)	161 ± 19.3 (11)^D^
		EC_80_	1030 ± 193 (11)	1045 ± 189 (11)	1612 ± 481 (9)	637 ± 118 (11)^E^
Viagra	U46619	EC_20_	8.00 ± 1.0 (10)	8.4 ± 2.6 (6)	8.3 ± 1.4 (7)	7.35 ± 1.5 (8)
(SC)		EC_50_	32.0 ± 3.9 (10)	33.7 ± 10.2 (6)	33.2 ± 5.5 (7)	29.4 ± 5.8 (8)
		EC_80_	128 ± 15.5 (10)	135 ± 41.0 (6)	133 ± 21.9 (7)	118 ± 23.2 (8)
	ACH	EC_20_	248 ± 96.8 (8)^A^	106 ± 25.2 (6)	67.1 ± 16.7 (6)	127 ± 33.6 (8)^B^
		EC_50_	990 ± 387 (8)^A^	425 ± 101 (6)	268 ± 66.6 (6)	509 ± 134 (8)^B^
		EC_80_	4329 ± 1767 (8)	1463 ± 466 (6)	1133 ± 277 (6)	2851 ± 1046 (8)^C^
	SNP	EC_20_	130 ± 45.4 (10)	88.9 ± 20.2 (6)	136 ± 36.8 (7)	179 ± 64.5 (7)^D^
		EC_50_	521 ± 181 (10)	356 ± 80.7 (6)	544 ± 147 (7)	715 ± 258 (7)^D^
		EC_80_	2748 **±** 1422 (10)	1955 **±** 651 (6)	3272 **±** 122 (7)	4781 **±** 2216 (7)^E^

All data are expressed as means ± SE with number of fetuses in parenthesis. All data for wild-type (WT)/P0 fetuses in control and sildenafil citrate (SC)-treated mice are expressed as effective concentration (EC in nM).

A Reduced ACH sensitivity in SC-treated WT male vs. control WT male (EC_20_, EC_50_; *P* < 0.05; Mann-Whitney *U*-test). ^B^ Reduced ACH sensitivity in SC-treated P0 female vs. control P0 female (EC_20_, EC_50_; *P* < 0.01, Mann-Whitney *U*-test). ^C^Reduced ACh sensitivity in SC-treated P0 female vs. control P0 female (EC_80_; *P* < 0.001, Mann-Whitney *U*-test). ^D^Reduced sodium nitroprusside (SNP) sensitivity in SC-treated P0 female vs. control P0 female (EC_20_, EC_50_; *P* < 0.01). ^E^Reduced SNP sensitivity in SC-treated P0 female vs. control P0 female (EC_80_; *P* < 0.05, Mann-Whitney *U*-test).

#### Effect of maternal SC treatment on vascular responses of fetal abdominal aortas.

There was no significant difference in abdominal aortic diameter between the four SC-treated groups; data were also comparable with controls (Kruskal-Wallis test; *P* > 0.05; Table 1). Abdominal aortic basal tone (0.9 of L_5.1kPa_ normalization) was not significantly different between the four SC-treated groups; data were also comparable with controls (Kruskal-Wallis test; *P* > 0.05; Table 1); these data corresponded to a basal tone for the whole group of 35 [17 − 67] mmHg [median (range; min to max); *n* = 31].

Contraction to each agonist (KPSS; 10^−5^ M PE; 2 × 10^−6^ M U46619) was not significantly different between each SC-treated experimental group (Kruskal-Wallis test; *P* > 0.05; Table 1). U46619 elicited greater agonist-induced contraction than PE (Kruskal-Wallis test; *P* < 0.05; Table 1). U46619-induced contraction was comparable in SC-treated vs. control animals (*P* > 0.05; two-way ANOVA; data not shown).

**Fig. 4.**
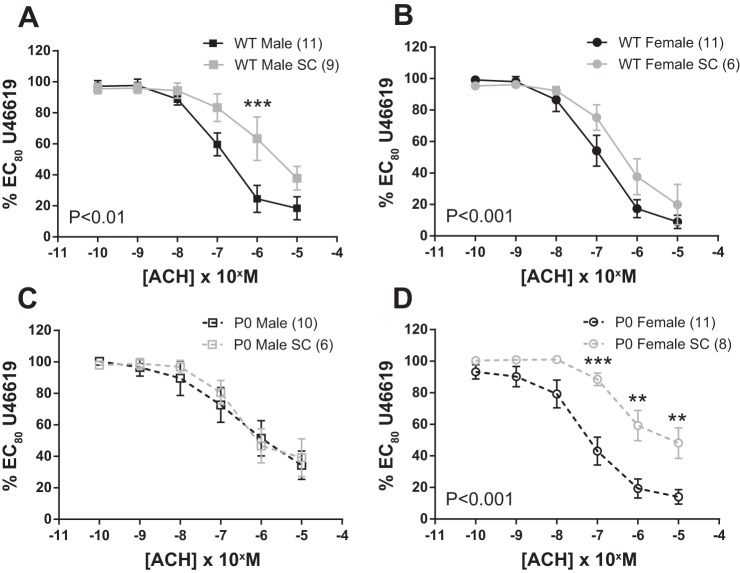
Relaxation of fetal abdominal aortas in response to increasing doses of ACh; comparisons between SC-treated and control dams. *A–D*: arteries were precontracted with EC_80_ dose of U46619. ACh dose-response curves were compared using two-way ANOVA followed by a Bonferroni post hoc test where appropriate. All data are means ± SE (*n* = number of animals). ■ denotes WT male, while ● denotes WT female, □ denotes P0 male, and ○ denotes P0 females. Fetuses were harvested from either SC-treated dams (gray) or control dams (black). Asterisks denote level of significance using Bonferroni post hoc test. **P < 0.01, ***P < 0.001.

ACh-induced relaxation was similar in SC-treated P0 male vs. control P0 male mice (*P* > 0.05; two-way ANOVA). However, in all other sex/genotype groups, SC treatment significantly blunted ACh-induced relaxation (WT male; *P* < 0.05, WT female and P0 female mice; *P* < 0.001; two-way ANOVA; Fig. 4).

**Fig. 5.**
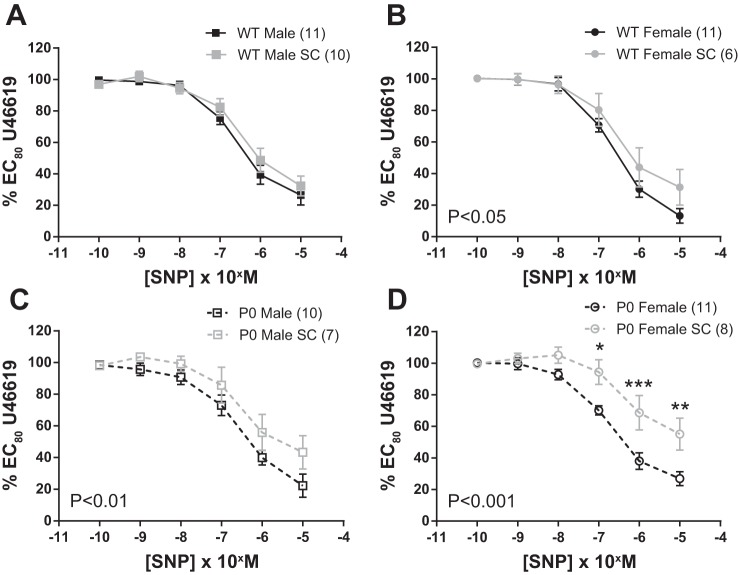
Relaxation of fetal abdominal aortas in response to increasing doses of SNP; comparisons between SC-treated and control dams. *A–D*: arteries were precontracted with EC_80_ dose of U46619. SNP dose-response curves were compared using two-way ANOVA followed by a Bonferroni post hoc test where appropriate. All data are expressed as means ± SE (*n* = number of animals). **P < 0.01. Fetuses were harvested from either SC-treated dams (gray) or control dams (black). Asterisks denote level of significance using Bonferroni post hoc test. *P < 0.05, **P < 0.01, ***P < 0.001.

SNP-induced relaxation was similar in SC-treated WT male vs. control WT male mice (*P* > 0.05; two-way ANOVA). However, in all other sex/genotype groups, SC treatment significantly reduced SNP-induced relaxation (P0 male; *P* < 0.01, WT female; *P* < 0.05 and P0 female mice; *P* < 0.001; two-way ANOVA; Fig. 5).

#### Effect of maternal treatment on fetal and placental phenotype.

Litter size, the number of resorptions, and the proportion of WT: P0 mice were not significantly affected by SC treatment dose used here (data not shown). As expected, and comparable to previous reports (2, 6, 8), P0 fetuses were significantly lower in weight compared with WT littermates (*P* < 0.05; Two-way ANOVA with Bonferroni post hoc test). Fetal and placental weights were not significantly affected by administration of the SC dose used here. As previously reported, placental weight was significantly increased in WT vs. P0 pups, with no effect of treatment (WT control: 103 ± 2 mg, WT SC: 107 ± 3 mg, P0 control: 77 ± 2 mg, P0 SC; 83 ± 4 mg. *P* < 0.001 for genotype, *P* > 0.05 for treatment; two-way ANOVA with Bonferroni post hoc test).

## DISCUSSION

The first aim of the study was to develop a methodology to assess fetal vascular function using wild-type mice and the P0 mouse model of FGR. We achieved this goal using wire myography to assess contraction and relaxation of isolated abdominal aortas.

We initially attempted to isolate and mount mesenteric arteries, an extensively used vessel subtype; arteries develop significant and sustained contractions in response to a number of agonists and reproducible relaxation to both endothelium-dependent and endothelium-independent agents (3, 12, 32). Unfortunately, although easily identified in E18.5 pups, mesenteric arteries were overly difficult to isolate intact. However, fetal abdominal aorta, a vessel used by a number of groups to study vascular function in adult control and knockout mice (e.g., 42, 47), proved an appropriate substitute.

Preliminary studies suggested that “classical” normalization damaged the abdominal aorta as seen with fetal umbilical arteries and veins (15). Therefore, we used a lower threshold (0.9 of L_5.1kPa_), as noted for human placental (4) and mouse umbilical (15) arteries and veins. This method produced a postequilibration median basal tone of 33 mmHg. Although there are no data on fetal aortic blood pressure in mice, this value is similar to that seen in human fetuses at a similar state of embryonic development; fetal aortic blood pressure was estimated to be 28 mmHg at 20 wk rising to 45 mmHg at 40 wk of gestation (41). Therefore, we are confident that using these normalization criteria and performing experiments in solutions gassed with 5% O_2_, we are observing vessels under physiological conditions.

With regard to vasoconstriction, application of depolarizing solution or agonist elicited significant contraction; similar responses to PE (47) and U46619 (42) have previously been documented in adult mouse aortic rings. As with mouse umbilical vessels (15), WT PE-induced contraction was significantly reduced compared with that with the thromboxane-mimetic U46619, supportive of previous studies, suggesting the latter activates vascular smooth muscle via calcium influx, calcium release from stores, and myofilament calcium sensitization (1).

U46619-induced contraction was significantly increased in WT males vs. WT females; similar data have been reported in adult rat aorta with PE and vasopressin (36). This may be a consequence of the humoral environment as Schror et al. (33) have demonstrated increased U46619-induced coronary artery contraction following testosterone administration in guinea pigs, and Higashiura et al. (11) have suggested differences in thromboxane receptor regulation and expression in isolated male and female rat aortic smooth muscle cells. This increased contraction in male vs. female aortic rings was not seen in P0 pups. U46619-induced contraction was reduced in P0 vs. WT males, whereas P0 vs. WT females showed identical contractile characteristics; however, in vivo umbilical artery Doppler ultrasound measures were not significantly altered in P0 and WT pups (6). Taken together, these data suggest a possible vascular defect in P0 male mice. The mechanism(s) for this reduced contraction requires further investigation.

The endothelium-dependent agonist ACh produced marked relaxation (<20% residual contraction), suggestive of a fully functioning, undamaged endothelium using this methodology. Similar reactivity has been reported in adult mouse abdominal aorta (19, 47). Data from animal models and humans suggest that endothelium-dependent relaxation is increased in females (see Ref. 27 for review). This pattern was seen in P0 mice, and there was a trend toward increased relaxation in WT females vs. WT males, but this did not reach significance (*P* = 0.29; two-way ANOVA). The lack of difference with ACh may be a consequence of the mechanism by which agonist-induced vasodilatation is achieved in this blood vessel subtype [i.e., the proportion of NO-, endothelium-derived hyperpolarizing factor- (EDHF), and prostaglandin-driven relaxation]. Alternatively, subtle differences in agonist-induced relaxation might only be apparent with an increased number of observations.

P0 females exhibited similar relaxation compared with WT females (Fig. 3). Maximal relaxation and agonist sensitivity (EC_50_) were also identical in both strains. The endothelium-independent agonist SNP produced similar relaxation in all groups, as has been reported in adult tissues (29).

Thus, we have clearly demonstrated the successful isolation and functional analysis of fetal abdominal aortas in a mouse model of FGR. This methodology is robust and sensitive enough to detect differences in vascular function with similar characteristics to data seen in adult vascular tissues.

The second aim of the study was to determine whether a maternally targeted treatment led to altered fetal physiology. As we wished to determine a proof of principle (i.e., our methodology would be able to detect the effect of a maternal treatment on fetal vascular reactivity), we increased the dosage of SC to maximize the chances of seeing altered abdominal aorta arterial function; SC dosage was increased to 0.8 mg/ml SC in drinking water using the same E12.5 to E18.5 dosing regimen, as previously observed (6). In the current study, SC administration did not significantly increase fetal weight in P0 or WT. In our previous study, a more clinically relevant SC dosage increased fetal weight in WT and P0 fetuses (6). These data suggest a biphasic effect of SC on fetal growth and, thus, care must be taken in balancing the dose of SC to produce optimal growth in pathological pregnancies. Indeed, previous ovine studies have suggested that SC treatment may lead to reduced uteroplacental perfusion when overall systemic vascular resistance is lowered (23).

Fetal abdominal aorta relaxation was diminished by this super-therapeutic SC treatment regimen in pregnant dams; ACh-induced relaxations were significantly reduced, except in P0 males, with the effect being more profound in female vs. male fetuses (Figs. 4 and 5). SC treatment also led to reduced SNP-induced relaxation, except in WT males, with the effect being most profound in the P0 females. Decreased SNP-induced (endothelium-independent) relaxation suggests desensitization of vascular smooth muscle cells to NO; similar data are seen in mouse models that overexpress eNOS (26). These mice have reduced vascular reactivity to NO through a reduced responsiveness to cGMP elevation. However, this high dose of SC is unlikely to be achieved in studies aimed at determining possible therapeutic potential of PDE-5 inhibitors to improve pregnancy outcomes. Importantly, significant ACh-induced abdominal aortic relaxation (>50%) was still noted in all groups; thus, other pathways (e.g., dilatory prostaglandins; EDHF-like responses) are still sensitive to agonist stimulation.

In summary, we have demonstrated the isolation and functional analysis of fetal abdominal aortas in a mouse model of FGR. This methodology is sensitive, detecting differences in vascular function between normally growing and FGR fetuses dependent on sex. Maternal treatment with a super-therapeutic dose of SC attenuated NO-stimulated relaxation of fetal abdominal aortas. We propose that this technical advancement represents a major step forward in our ability to assess the effect of maternal treatment strategies on vascular function of the offspring in mouse models of pregnancy disease. Combining this technique with in vivo Doppler ultrasound measurements in mouse models will be particularly useful for efficacy and safety profiling for maternal treatments of FGR/PE.

## DISCLOSURES

No conflicts of interest, financial or otherwise, are declared by the authors.

## AUTHOR CONTRIBUTIONS

Author contributions: L.J.R., M.R.D., S.L.G., C.P.S., and M.W. conception and design of research; L.J.R. and M.W. performed experiments; L.J.R. and M.W. analyzed data; L.J.R., M.R.D., S.L.G., and M.W. interpreted results of experiments; L.J.R. and M.W. prepared figures; L.J.R., S.L.G., C.P.S., and M.W. drafted manuscript; L.J.R., M.R.D., S.L.G., C.P.S., and M.W. edited and revised manuscript; L.J.R., M.R.D., S.L.G., C.P.S., and M.W. approved final version of manuscript.
